# Patterns-of-Care Analysis for Radiotherapy of Elderly Head-and-Neck Cancer Patients: A Trinational Survey in Germany, Austria and Switzerland

**DOI:** 10.3389/fonc.2021.723716

**Published:** 2022-01-03

**Authors:** Erik Haehl, Alexander Rühle, Simon Spohn, Tanja Sprave, Eleni Gkika, Constantinos Zamboglou, Anca-Ligia Grosu, Nils H. Nicolay

**Affiliations:** ^1^ Department of Radiation Oncology, University of Freiburg - Medical Center, Freiburg, Germany; ^2^ German Cancer Consortium (DKTK) Partner Site Freiburg, German Cancer Research Center (dkfz), Heidelberg, Germany

**Keywords:** HNSCC (head and neck squamous cell carcinoma), elderly patients, radiotherapy, chemotherapy, patterns-of-care

## Abstract

**Objectives:**

The number of elderly head-and-neck squamous cell carcinoma (HNSCC) patients is increasing, and clinical trials defining the standard of care either excluded or underrepresented elderly patients. This leaves physicians with a challenging and highly individual treatment decision largely lacking clinical evidence.

**Methods:**

A tri-national patterns-of-care survey was sent to all members of the German (DEGRO), Austrian (ÖGRO), and Swiss (SRO/SSRO) national societies of radiation oncology. The online questionnaire consisted of 27 questions on the treatment of elderly HNSCC patients, including 6 case-based questions. Frequency distributions and subgroup comparisons were calculated using SPSS statistics software.

**Results:**

A total of 132 answers were collected, including 46(35%) form universities, 52(39%) from non-university-hospitals and 34(26%) from private practices. 83(63%) treat 1-5 and 42(32%) >5 elderly HNSCC patients per month. Target volumes are defined analog current guidelines by 65(50%) of responders and altered based on age/comorbidities or tumor stage by 36(28%) and 28(22%), respectively. Chemotherapy is routinely administered by 108(84%) if indicated, with weekly 40mg/m^2^ of cisplatin being the favored regimen by 68(53%) in the definitive situation and 60(47%) in the adjuvant setting. Hypofractionation and hyperfractionation/acceleration are used by 26(20%) and 11(9%), respectively. Only 7(5%) clinicians routinely recommend inpatient treatment for elderly HNSCC patients. In a typical definitive patient case, 73(63%) responders recommended chemoradiation with bilateral elective node irradiation analog current guidelines. In an adjuvant example case recommendations regarding elective volume and chemotherapy were heterogeneous. Differences between responders’ institutions concern the frequency of PET-CT in staging, preventive port-catheter and PEG implantation, the choice of chemotherapy regimens and the use of alternative fractionations.

**Conclusion:**

Treatment of elderly HNSCC-patients in the German-speaking countries mainly follows guidelines established for younger patients. Algorithms for patient stratification and treatment de-escalation for “unfit” elderly patients are needed.

## Introduction

The incidence of head-and-neck squamous cell carcinoma (HNSCC) in elderly patients is rising (1). In the Western world, almost a quarter of HNSCC patients are above 70 years with a further increase prognosticated due to ongoing demographic changes (2, 3).

Current treatment standards comprise surgery, followed by adjuvant (chemo-)radiation for locoregionally advanced cancers, or primary (chemo)radiation. Limited data are available that define the optimal treatment approach for elderly HNSCC patients. The landmark trials defining the role of radiotherapy for HNSCC excluded or underrepresented elderly patients (4–7), but the available data suggest comparable efficacy of radiotherapy despite reduced benefit from concomitant chemotherapy or altered fractionation schemes (8–14). While primary radiotherapy spares vulnerable patients invasive tumor treatments, it often results in significant and sometimes severe acute and chronic toxicities (10, 13, 15). Higher-grade toxicities may be especially problematic in elderly and vulnerable patients that suffer from comorbidities and an already reduced quality-of-life.

Consecutively, demographic studies have shown that the probability of elderly HNSCC patients to receive curative treatment is considerably lower than that of younger patients (14, 16). Irrespective of existing comorbidities, age has been reported as an independent factor for non-standard treatment (16). However, retrospective data yielded similar resulting quality-of-life in elderly HNSCC patients receiving curative treatments in comparison with younger patients and suggest that aggressive and curative treatments may be feasible also in the elderly (17, 18).

It has to be considered that elderly patients favor a preservation of their quality-of-life over a pure benefit in overall survival, diminishing the acceptance of aggressive cancer treatments (19, 20). Additionally, elderly patients are more likely to die from non-cancer deaths as a manifestation of their age-related comorbidities (8, 21).

These aspects leave treating physicians with challenging and highly individual therapeutic decision making for this distinct patient cohort, lacking support of evidence-based guidelines and a need for harmonization of treatment recommendations. This trinational pattern-of-care survey aimed to analyze the real-world treatment pathways of elderly HNSCC patients in Germany, Switzerland and Austria.

## Methods

### Questionnaire

An anonymous online questionnaire was created consisting of 27 questions. 21 questions were multiple choice, 6 allowed for multiple responses including individual answers. The first part of the questionnaire was a patterns-of-care survey consisting of three questions regarding supplier information, three questions on pretreatment procedure and 15 questions on therapy parameters. The second part was a case-based survey of two typical HNSCC scenarios in a definitive and adjuvant radiotherapy setting. Each case presented three multiple-choice questions regarding general treatment approach including concomitant chemotherapy and elective nodal volume, prescribed doses and fractionation scheme.

### Distribution and Response Collection

The questionnaire was made accessible through the website of a service software company (SurveyMonkey, San Mateo, CA, USA). A copy of the questionnaire can be found in the appendix (**Supplementary Table 1**). The invitation link to the survey was sent *via* email to all members of the German (DEGRO), Austrian (ÖGRO), and Swiss (SRO/SSRO) national societies of radiation oncology to get a comprehensive representation of the patterns of care in German-speaking countries. The survey was open from September 30^th^ to November 15^th^, 2020. Replies were recorded, stored and analyzed anonymously.

### Statistics

Frequency distributions were calculated and visualized using pie charts and bar diagrams. For subgroup comparisons, Pearson’s chi-squared tests were used. A *p*-value below 0.05 was considered statistically significant throughout the study. All statistical analyses were carried out with SPSS software, version 27 (IBM, Armonk, NY, USA).

## Results

### Interview Cohort

**Figure 1:**
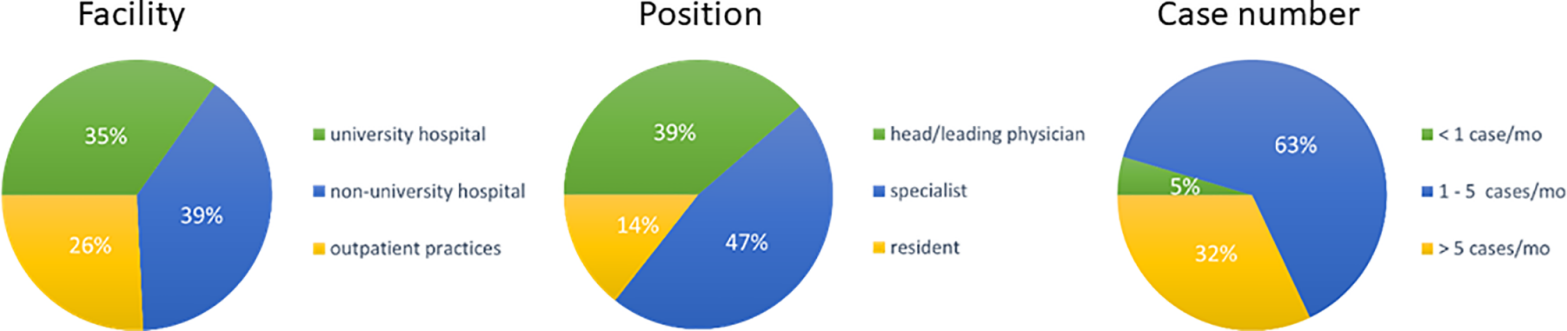
Pie charts for the type of responders’ facility (n=132), responders’ position (n=132) and treated cases per month (n=) indicated as percentage of responders. (mo, months).

A total of 1885 inquiries were sent, of which 132 were answered (7%). Most responses were received from non-university hospitals (n=52, 39%), followed by 46 (35%) from university hospitals and 34 (26%) from private radiation oncology practices. 86% (n=116) of responders were radiation oncology specialists, with a high proportion of 39% (n=51) being head physicians or chief senior physicians, and 14% (n=19) being radiation oncology residents. Expertise in treating elderly HNSCC patients was generally high, with 63% (n=83) of responders treating 1-5 cases monthly and 32% (n=42) treating more than 5 cases per month. As expected, responders from university hospitals and non-university hospitals reported significantly higher case numbers than patients from private outpatient radiation oncology practices (51% and 31% vs 9%, *p*=0.002) (**Figure 1**).

### Patterns of Tumor Staging and Patient Assessment in Elderly Head-and-Neck Cancer Patients

**Figure 2:**
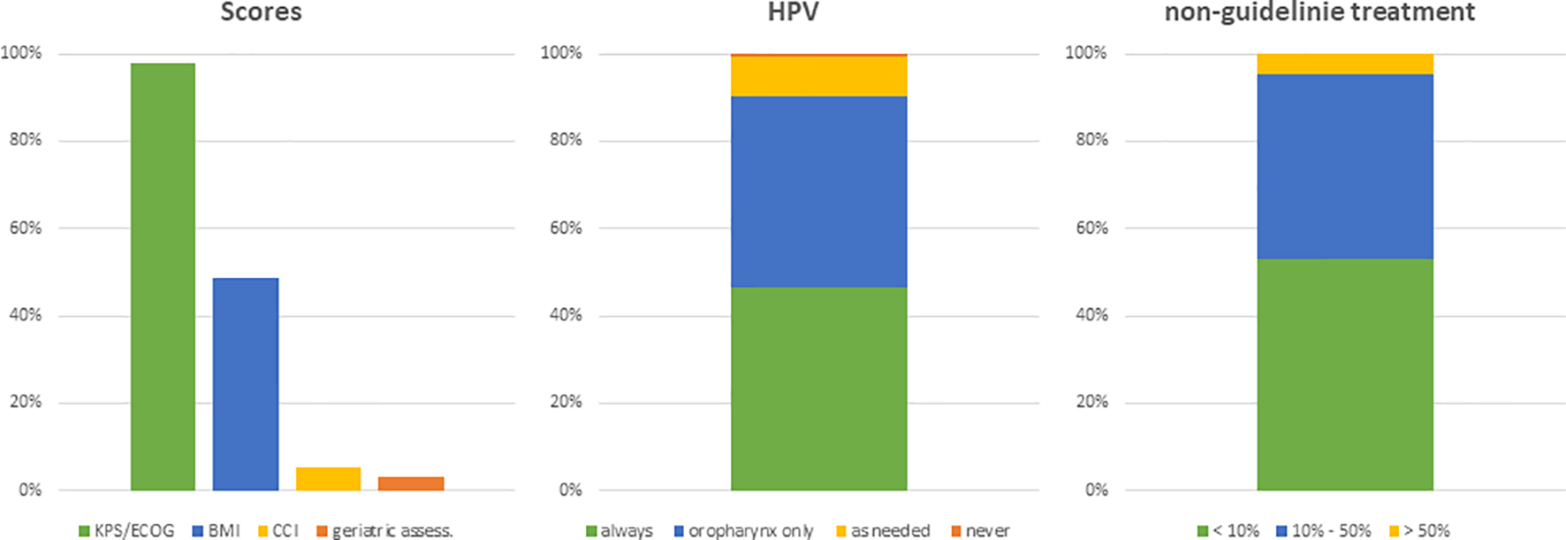
Bar charts indicating the usage of different scores in patient assessment (n=131), the evaluation of the HPV status (n=130) and the assumed proportion of patients not treated based on current guidelines (n=128). (KPS, Karnofsky performance status; BMI, body mass index; CCI, Charlson Comorbidity Index; HPV, human papillomavirus).

**Figure 3:**
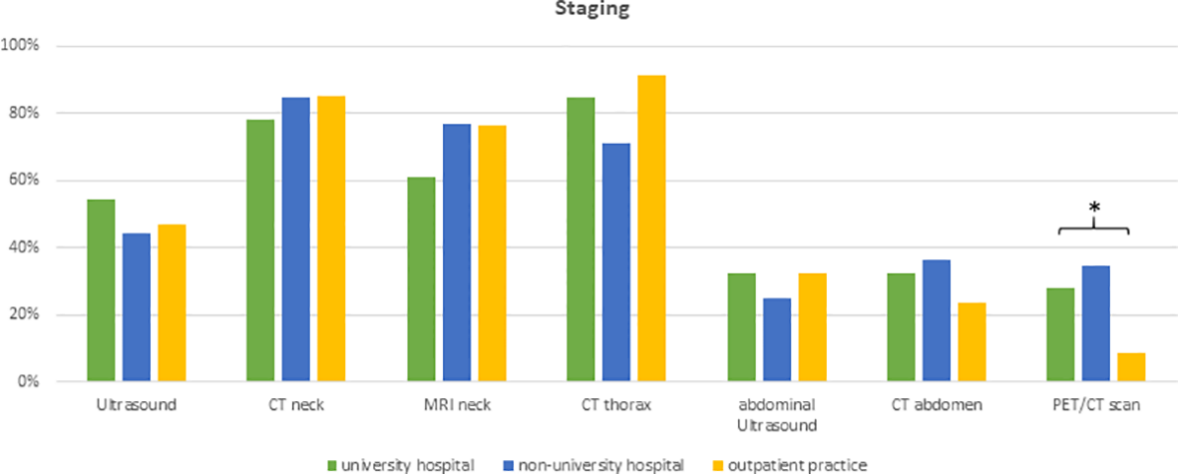
Bar charts indicating the usage of different imaging modalities in staging, separated for responders’ type of facility (n=131). * indicating *p*<0.05 Pearson’s chi-squared test. (CT, computed tomography; MRI, magnetic resonance imaging; PET, positron emission tomography).

The first set of questions aimed at investigating the patterns for geriatric patient assessment and pre-treatment tumor staging. 98% of physicians (n=128) reported to include performance status in their routine clinical assessment and 49% (n=64) assessed the body mass index prior to radiotherapy initiation. Only a small minority of radiation oncologists conducted structured comorbidity evaluations such as the Charlson Comorbidity Index (n=7, 5%) or a routine geriatric assessment (n=4, 3%). 48% (n=63) of radiation oncologists reported that they routinely analyze HPV status irrespectively of the tumor localization and another 42% (n=54) only for oropharyngeal cancers. The frequency of HPV assessment did not differ between university hospitals, non-university hospitals or private practices (*p*=0.36) (**Figure 2**). Pre-treatment staging relied on cross-sectional imagining of the head-and-neck region in all responses (100%, n=131) (CT: n=109, 83%; MRI: n=94, 71%) and the thorax (CT thorax 82%, n=107) without significant differences between types of treating facilities. University hospitals and non-university hospitals were significantly more likely to use PET-CT for staging compared to outpatient practices (n=13 (29%) and n=18 (35%) vs n=3 (9%), *p*=0.009) (**Figure 3**).

### Treatment Patterns in Elderly Head-and-Neck Cancer Patients

**Figure 4:**
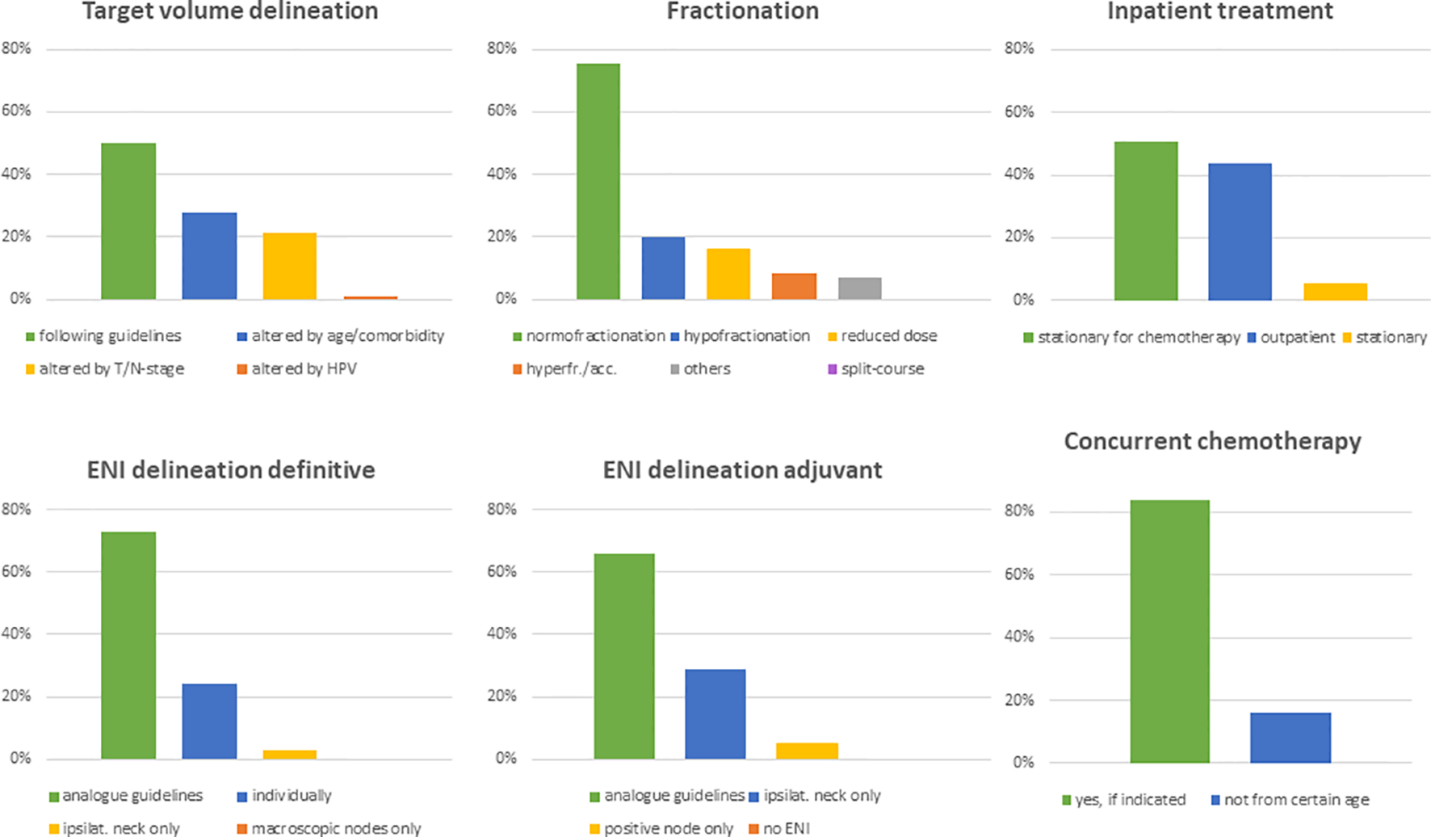
Bar charts indicating alteration of target volumes (n=130), fractionation schemes (n=130), treatment modality (n=130), elective nodal volumes in the definitive (n=129) and adjuvant setting (n=129) as well as the application of concomitant chemotherapy in elderly HNSCC patients (n=129). (ENI, elective nodal irradiation).

The next set of questions addressed the radiation treatment of elderly HNSCC patients. 53% (n=68) of physicians claimed to treat less than 10% of elderly patients outside of guidelines or recommendations, and another 42% (n=54) reported that they deviated from current treatment guidelines in 10 to 50% of cases. 50% (n=65) of physicians routinely used guideline-based target volume definitions irrespectively of the patient age, while 28% (n=36) reported to adapt target volume definition in dependency of age and comorbidities and 22% (n=28) in dependency of the tumor stage (**Figure 4**).

For delineation of the elective nodal volume in definitive radiation treatments, 73% (n=97) of radiation oncologists based their volumes on established consensus guidelines and 24% (n=35) individually modified it routinely. In the adjuvant setting, only 66% (n=85) of physicians stated that they follow consensus guidelines, whereas 29% (n=37) spared the uninvolved neck side and 5% (n=7) of responders only irradiated pathologically affected lymph node levels. 75% (n=97) of replying physicians indicated that they use a normofractionated schedule in the treatment of elderly HNSCC patients. Hypofractionation, dose reduction and hyperfractionation/acceleration was routinely used by 20% (n=26), 16% (n=21) and 8% (n=11) of health-care providers, respectively. University hospitals and non-university hospitals reported to use hypofractionation and hyperfractionation/acceleration significantly more often compared to outpatient practices (n=10 (22%) and n=14 (28%) vs n=2 (6%), *p*=0.019 for hypofractionation; n=7 (16%) and n=4 (8%) vs 0 (0%), *p*=0.041 for hyperfractionation/acceleration) (**Figure 4**). Radiotherapy is applied in an outpatient setting by 95% (n=123) of physicians if medically reasonable. 51% (n=66) of radiation oncologists stated that they hospitalize patients only for chemotherapy. Only 5% (n=7) of responders generally recommended inpatient radiotherapy treatment for elderly HNSCC patients (**Figure 4**). Treatment monitoring usually consists of weekly visits in the outpatient setting (n= 90, 69%).Only one responding physician used divergent dose constraints for organs at risk in elderly HNSCC patients, namely for the parotid gland. Generally, the vast majority does not de-escalate radiotherapy treatment for HPV-positive HNSCCs in elderly patients (n=111, 85%). However, 18% (n=8) of university physicians include those patients in de-escalation trials.

**Figure 5:**
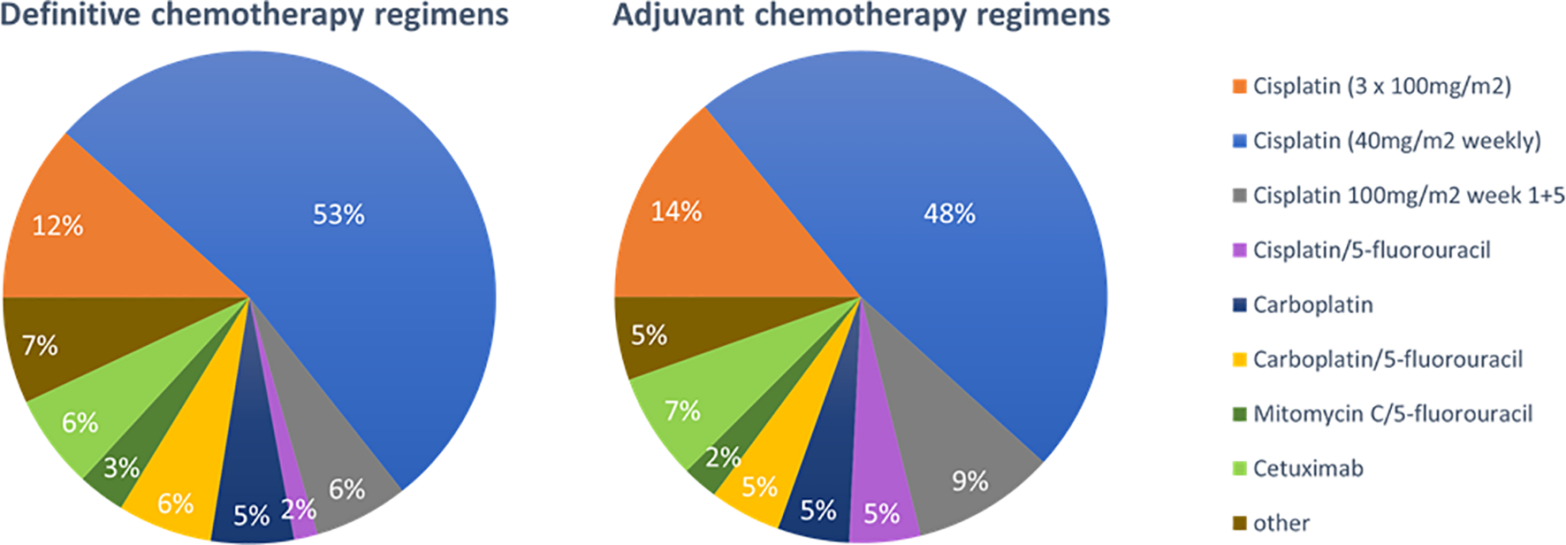
Pie charts for the preferred concomitant chemotherapy regimens in the chemoradiation of elderly HNSCC patients in the definitive (n=129) and adjuvant (n=128) setting.

The role of concomitant chemotherapy was considered more controversial. Although 84% (n=108) of physicians stated to routinely prescribe chemotherapy following the established indications for younger patients, at least 16% (n=21) reported a defined chronological age cut-off for chemotherapy ranging at 75 years in median (range 65-80 years) (**Figure 4**). For individual treatment decisions regarding concomitant chemotherapy, a variety of parameters were considered including performance status (n=129, 99%), blood parameters (n=128, 98%), age (n=106, 81%), cardiac function (n=77, 68%), hearing (n=89, 59%) and comorbidities (n=26, 20%). In the free text answers, the expected patient compliance was suggested as additional decision aid by some responders. Platinum-based chemotherapy was the standard regimen in our patterns-of-care analysis (n=112, 87%). The most common regimens in the definitive setting were weekly 40mg/m^2^ cisplatin (n=65, 53%), three cycles of 100mg/m^2^ (n=15, 12%) cisplatin, two fractionated cycles of 100mg/m^2^ cisplatin in weeks 1 and 5 (n=9, 6%), carboplatin/5-FU (n=8, 6%) and cetuximab (n=8, 6%). A broad variety of other regimens were used infrequently (**Figure 5**).

Concerning adjuvant therapy, the used chemotherapy regimens were comparable with slight changes towards a higher use of cisplatin/5-FU [n=2 (2%) vs n=6 (5%)] and fractionated cisplatin in weeks 1 and 5 (n=8 (6%) vs n=13 (9%)) (**Figure 5**). In the definitive as well as in the adjuvant setting, distribution of chemotherapy regimens differed between supplier institutions: Physicians in university hospitals more often prescribed cisplatin 3x100mg/m^2^ compared to non-university hospitals and outpatient practices (n=10 (22%) vs n=4 (8%) and n=3 (3%), *p*=0.018 in the definitive setting; n=11 (25%) vs n=5 (10%) and n=2 (6%), *p*=0.002 in the adjuvant setting).

**Figure 6:**
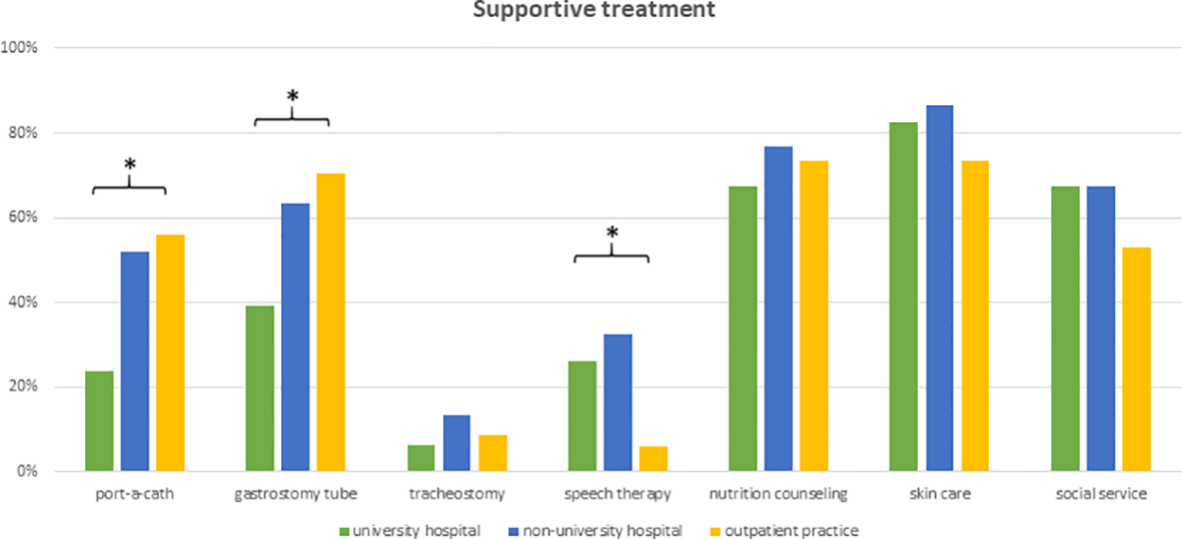
Bar charts indicating the application of supportive interventions routinely used in the treatment of elderly HNSCC patients, separated for responders’ type of facility (n=129). * indicating *p*<0.05 Pearson’s chi-squared test.

Physicians reported a wide variety of supportive therapies during radiotherapy of elderly HNSCC patients. Skincare and nutrition counseling were offered as standard of care by 84% (n=108) and 74% (n=96) of responders, respectively. Social service support was routinely offered by 65% (n=84) of health-care providers. Prophylactic placement of feeding tubes or port catheters was standardly ordered by 58% (n=75) and 44% (n=57) of responding physicians. University hospital-based physicians were significantly less likely to initiate prophylactic feeding tubes (*p*=0.004) or port catheter implantation (*p*=0.009). Routine logopedic training and prophylactic tracheotomy were routinely recommended only by 24% (n=31) and 10% (n=13) of physicians, respectively (**Figure 6**).

Follow-up care in the elderly was stated to be identical to younger patients (n=116, 91%). Only 7% (n=9) of responders reported a shorter follow-up interval in elderly HNSCC patients.

### Representative Patient Cases

**Figure 7:**
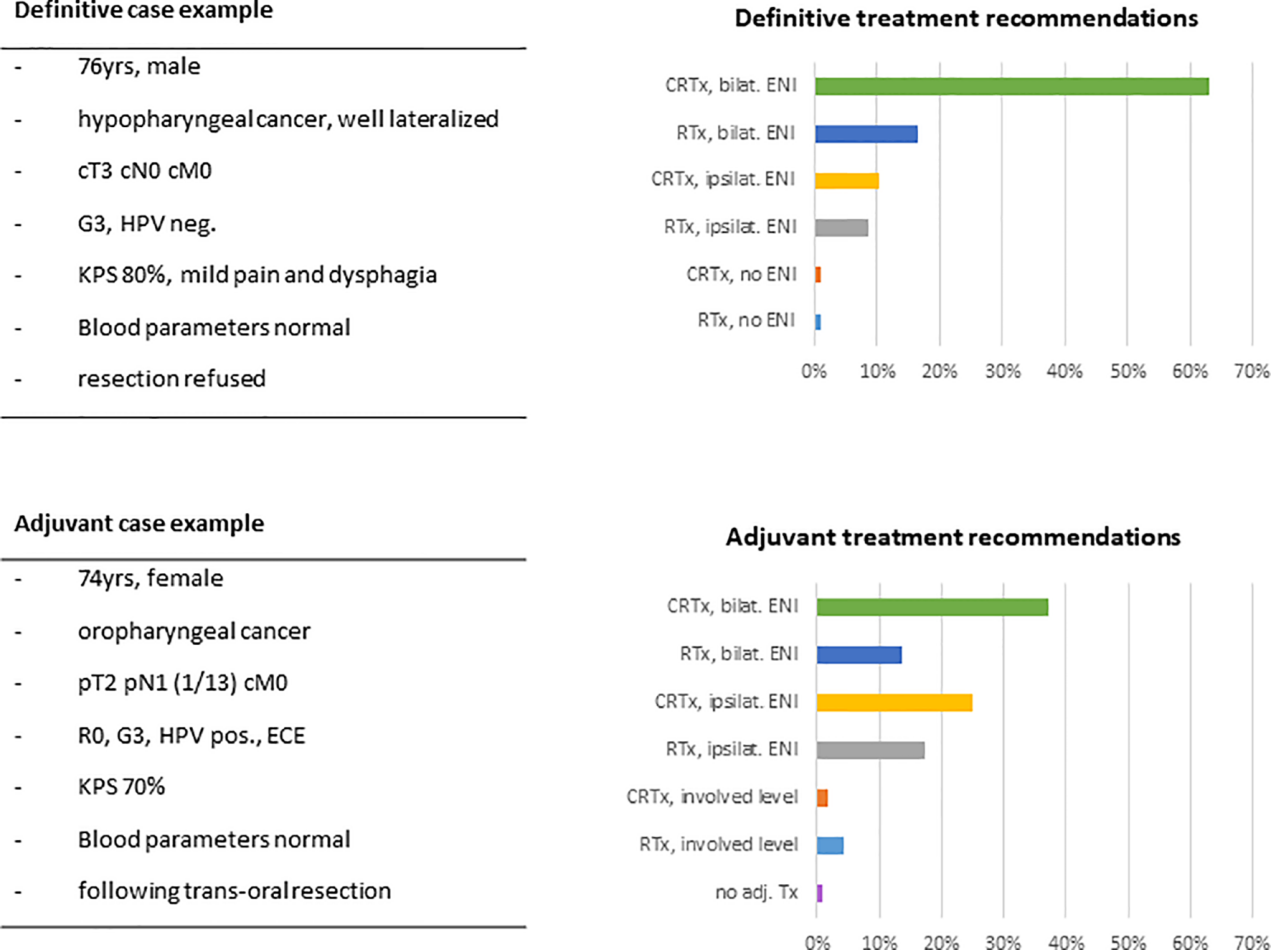
Example case of a typical elderly HNSCC patient in the definitive and in the adjuvant setting with key features and suggested treatment approaches given in percentage of responders (n=116). (HPV, human papillomavirus; KPS, Karnofsky performance status; CRTx, chemoradiation; RTx, radiotherapy; ENI, elective nodal irradiation; ECE, extracapsular extension.

The first case presented a typical definitive HNSCC setting of a 76-year-old patient with a locally advanced but non-metastatic tumor, where resection was declined by the patient. Asked about the general treatment approach, most responders recommended definitive chemoradiation including bilateral elective nodal volumes (n=73, 63%). 26% (n=30) would omit chemotherapy and 19% (n=22) would spare the contralateral neck. Dose prescription to the primary tumor would be 70Gy (EQD2) by 79% (n=92) of responders; 18% (n=21) of physicians would prescribe higher and only 3% (n=4) lower doses. 89% (n=105) of radiation oncologists would use conventional fractionation. Recommendations for acceleration, hyperfractionation and hypofractionation were given by 8% (n=9), 4% (n=5) and 3% (n=4) of responding physicians, respectively (**Figure 7**).

The second case presented a 74-year-old patient after complete resection of an HPV-associated oropharyngeal carcinoma with pathologically ascertained extracapsular spread of the resected lymph node metastasis. In this setting, radiation treatment recommendations of our responders were more heterogeneous. 37% (n=43) of radiation oncologists would favor adjuvant chemoradiation including the bilateral neck. Chemotherapy would be omitted by 35% (n=41) of physicians. 48% (56) of responders would spare the contralateral neck, with 12% (n=7) of those limiting lymph node irradiation to the involved lymph node level. Only one responder (1%) would completely omit adjuvant therapy. Dose recommendations were given at 66Gy and 60Gy by 47% (n=55) and 42% (n=49) of physicians, respectively. Doses of ≥70Gy were recommended by 8% (n=10) of responders, and 3% (n=3) would prescribe less than 60Gy. 98% (n=115) of physicians would choose conventional fractionation (**Figure 7**).

## Discussion

We conducted a trinational patterns-of-care-survey for elderly HNSCC patients, that assesses the execution of radiotherapy in this distinct patient cohort. In summary, we observed high concordance of the treatment of elderly HNSCC patients with current guidelines for younger patients. However, there were several aspects that considerably varied between the participating radiation oncologists, for instance, chemotherapy administration, contralateral elective lymph node irradiation especially in the adjuvant setting and prophylactic feeding tube or port catheter implantation. An EORTC led survey conducted in 2018 showed as well significant heterogeneity between treating institutions and countries and concluded with a need for a consensus-based guideline for elderly HNSCC patients; to whose establishment this survey may contribute (22).

The elderly patient is not conclusively defined, yet classifications usually start at ages between 65 and 70 years. The United States National Institute of Aging consensus definition subdivides between “young old” (65-74 years), “older old” (75-84 years) and “oldest old” (≥ 85 years) (23). Recent literature often extends this by the so-called nonagenarian patient older than 90 years (24, 25). The clinical implication of this arbitrary classification is uncertain. Apart from some disease-specific etiological differences in elderly HNSCC patients, namely the higher prevalence of female patients and the lower alcohol and tobacco intake, most obstacles in the management of elderly patients seem to be associated with age itself (26). Of those, the influence of comorbidity is probably the best-studied parameter for oncological outcome in elderly patients. A recent US-population based study for example showed the high prevalence of hypertension, COPD, diabetes and cardiac disease in elderly HNSCC patients (27), and many authors described a strong correlation between comorbidity burden and reduced survival for elderly HNSCC patients, which is mainly related to more non-cancer related deaths of elderly patients (28–30). Some studies even demonstrate worse cancer specific outcome in patients with a high burden of comorbidity (30). This could either be caused by less aggressive treatment in those patients or non-cancer death misattributed to cancer (14, 31, 32). More importantly functional impairment and comorbidity together result in significant non-cancer related deaths of elderly patients. The MACH-NC analysis, the largest meta-analysis analyzing chemoradiation in HNSCC patients, reported a non-cancer mortality of 39% at a median follow-up of 5.6 years in patents aged above 70 years, which is significantly higher than in younger patients with 15% at the age of 50 years (8).

In addition, elderly patients are assumed more vulnerable to treatment-related toxicities, although results on this question are heterogeneous. Van der Walde et al. found multiple conflicting retrospective studies stating either increased or similar radiotherapy-induced toxicity in elderly patients compared with their younger peers (14). In a large monocentric retrospective study, we previously reported low chronic toxicity albeit significant acute toxicity (10). The correlation between increased toxicity and the addition of chemotherapy in elderly patients however is more established (14, 33, 34). Those considerations result in a significantly reduced probability of elderly HNSCC patients to receive curative treatment (14, 16). A Dutch study for example states that only 60% of HNSCC patients above 70 years receive standard treatment according to current guidelines (16), falling in line with the results of our survey. This emphasizes the need for development of evidence-based de-escalation Strategies, since elderly patients are reported to favor a preservation of their quality-of-life over pure benefit in overall survival, thus reducing the acceptance of aggressive cancer treatment (19).

In contrast, early and more recent recommendations refrain from generally excluding the elderly patient from the intensified curative treatment approach for HNSCC (2, 12). Radiotherapy seems to be feasible even in the oldest olds and retrospective studies suggest respectable local control rates or even similar survival rates to younger patients (10–12, 35, 36).

The role of chemotherapy is more debatable and evidence is strong to spare elderly patients the increased toxicity coming along with it. The MACH-NC meta-analysis showed no survival benefit for patients older than 70 years and only little effect for those from 61-70 years in case of concomitant application of chemotherapy in the definitive setting (8). This observation has been backed up by several retrospective analyses (10, 37). However, recent data reported similar survival to younger patients after chemoradiation in elderly patients with low comorbidities and high performance status (33, 38). In the postoperative setting, platinum-based chemoradiotherapy is established standard of care for the high risk factors extracapsular lymph node spread and positive resection margin. Unfortunately, both landmark studies did not involve a significant number of patients older than 70 years (5, 6, 39). In three more recent retrospective analyses, only one showed a small benefit of adjuvant chemoradiotherapy in elderly patients with high nodal tumor burden (40, 41). In our survey, the majority of healthcare providers did not set a strict age limit for the concomitant administration of chemotherapy. This should be critically reevaluated in the presence of available literature.

Concerning the chemotherapy agent, cisplatin is the standard of care for head and neck cancers in the definitive setting (4, 8). A prospective study testing weekly 30mg/m^2^ versus the current standard of 3x100mg/m^2^ found the latter to be superior albeit higher toxicity (42). Noteworthy, this study was criticized for an insufficient total cisplatin dose < 200mg/m^2^ in the weekly arm. For the adjuvant setting weekly cisplatin with 40mg/m^2^ has recently proven non-inferiority and to be of less toxicity (43). However, neither of these studies stratified for age. In our patterns-of-care analysis, weekly cisplatin with 40mg/m^2^ was the most widely used chemotherapy regimen both in the definitive and in the adjuvant setting. While this corresponds with the standard-of-care in the adjuvant setting, it is presumably a compromise between the efficacy of high-dose cisplatin and the lower toxicity and better controllability of weekly application schemes in the definitive setting (44).

Alternative fractionation schemes as an alternative to chemoradiation have shown little effectiveness in elderly patients (9, 45). Accordingly, the responders of our survey rarely use them in their routine. Hypofractionation was employed by a significant proportion of treating clinicians in our survey. In the literature, a variety of hypofractionated regimes has been reported ranging from semi-curative doses to sole palliation. Laursen et al. investigated 56Gy in 14 fractions (EQD2 of 65Gy) in an elderly patient cohort with a median of 74 years with good tumor response and acceptable tolerability contrasting with the ‘Quad shot’ regimen (one to three cycles of 14Gy in four fractions given twice daily for two consecutive days) offering effective palliation with minimal toxicity (46, 47). A recent review summarized common hypofractionation regimens for elderly patients and concluded that the choice of the correct regimen remains a highly individual decision (48).

De-escalation strategies, on the other hand, are largely lacking high-level evidence. The subpopulation of patients with HPV-associated tumors, featuring better prognosis, has been repeatedly investigated for potential treatment de-escalation strategies. This is matched by the heterogeneous recommendations regarding treatment de-intensification in our second case example. It has to be noted that cetuximab-based bio-radiotherapy failed to demonstrate non-inferiority or reduced toxicity compared to cisplatin-based chemoradiation (7, 49–51), and other de-escalation strategies still have to prove their clinical safety (52, 53). Responders of our study already report relatively high HPV testing that could be beneficial as de-escalation strategies get more established. Other biological markers such as tumor-infiltrating lymphocyte levels or tumor hypoxia have been shown to be prognostic but still require further evaluation prior to incorporation into potential treatment personalization strategies (54, 55). Clinical parameter like performance status, although widely used in the clinical routine, are not fully reliable for individualizing treatment due to their high inter- and intra-observer variability (56). Our group recently suggested a novel and validated prognostic score for elderly HNSCC patients based on KPS, Charlson Comorbidity Index and baseline CRP as a more accurate basis for treatment personalization (57).

It is commonly agreed that the chronological age is an insufficient guide for individual cancer management and the chronological definition of an elderly patient is to some extent arbitrary (2, 58). But the silver lining of a biological age lacks a consistent definition and is far from entering clinical routine. Patterns of DNA methylation, telomere shortening or blood marker panels have been suggested (59–61) but their role in cancer patients is completely unknown. Besides molecular definitions of biological age, functional age addresses the distinct multifaceted impairments associated with age such as frailty, reduced mobility, difficulties in social participation and maintaining of purpose, decline in cognitive function and the burden of comorbidities (62, 63). As a diagnostic tool, geriatric assessment (GA) has been introduced into the oncologic decision-making process (64–66). It encompasses important health domains as well as social and cognitive dimensions and therefore provides a more holistic assessment of a patient’s functional or fitness levels. As comprehensive GA is time- and resource-consuming, they were rarely reported to be used in our survey; however, many screening tools have been developed. G8, IADL and others already showed prognostic value for long-term quality-of-live, survival and vulnerability to toxicity in elderly HNSCC patients (67–69). Ongoing trials prospectively evaluate the value of GA screening tools and even stratify and de-escalate treatment for elderly patients in dependence of a full GA; however, results of these studies are pending (70, 71). Besides information about prognosis and its value for treatment decision, GA allows individually tailoring supportive interventions. Feasibility of screening and allocation pathways have already been established (72).

Our study faces certain limitations. The response rate to our survey was low albeit similar to comparable surveys conducted among members of the German Society of Radiation Oncology (DEGRO) (73, 74). Surveys addressing single institutions within the DEGRO achieved higher response rates but comparable absolute response counts (75, 76). In large centers, it is conceivable that the survey was only answered by the specialist in charge of treating elderly head-and-neck cancer patients. Some colleagues from private practices may lack experience with head-and-neck cancer treatments as it requires a large interdisciplinary infrastructure and thus, they may not have contributed to our survey. For data analysis of our survey, all forms were quantified equally. As a consequence, the share of patients treated by radiation oncologists that treat large numbers of elderly HNSCC patients could be underrepresented. However, university hospitals were overrepresented in our survey compared to small out-patient practices. We therefore think that our survey adequately covers the large number of patients treated in specialized centers. Due to the assured anonymization of response collection, the number of participating institutions could not be evaluated. Last, answers were not verified with clinical or demographic data and thus may be susceptible to a recall bias of the responders.

Despite these limitations, this tri-national survey gives valuable insights into the treatment patterns of the challenging cohort of elderly HNSCC patients. It shows a need for patient stratification algorithms to identify those patients profiting from aggressive treatment on the one side and those benefiting from de-escalation strategies on the other side.

## Data Availability Statement

The raw data supporting the conclusions of this article will be made available by the authors, without undue reservation.

## Author Contributions

EH and NN: Study concept and study design. EH and NN: Data acquisition, data analysis and data interpretation. EH and NN: Statistical analysis, manuscript preparation, manuscript editing. EH, AR, SS, TS, EG, CZ, ALG, NN: Manuscript reviewed. All authors contributed to the article and approved the submitted version.

## Conflict of Interest

The authors declare that the research was conducted in the absence of any commercial or financial relationships that could be construed as a potential conflict of interest.

## Publisher’s Note

All claims expressed in this article are solely those of the authors and do not necessarily represent those of their affiliated organizations, or those of the publisher, the editors and the reviewers. Any product that may be evaluated in this article, or claim that may be made by its manufacturer, is not guaranteed or endorsed by the publisher.
