# Supplementary material for: Patterns-of-Care Analysis for Radiotherapy of Elderly Head-and-Neck Cancer Patients: A Trinational Survey in Germany, Austria and Switzerland
Source: Front Oncol. 2022 Jan 3;11:723716. doi: 10.3389/fonc.2021.723716 (PMC8761738; doi:10.3389/fonc.2021.723716)
Supplement: Supplementary file 1 [file Table_1.docx]

| Questionnaire |
| --- |
| Supplier information |
| - - - 1. In what kind of institution do you currently work? |
| - - - 1. What is your current position? |
| - - - 1. How many elderly (> 70 years) HNSCC patients do you treat in your institution per month? |
| Preparation |
| - - - 1. What scores and classifications do you assess routinely for elderly (> 70 years) HNSCC patients? |
| - - - 1. What staging imaging studies do your routinely perform for elderly (> 70 years) HNSCC patients? |
| - - - 1. In what situations do you examine the HPV-status of elderly HNSCC patients in your institution? |
| Therapy |
| - - - 1. Do you de-escalate therapy for HPV-positive tumors in elderly (> 70 years) HNSCC patients (i.e. target volume reduction or reduced dose)?       2. Do you adapt target volume definition for elderly (> 70 years) HNSCC patients? |
| - - - 1. What part of the elective nodal volumes are you routinely irradiating in the definitive setting in elderly (> 70 years) HNSCC patients? |
| - - - 1. What part of the elective nodal volumes are you routinely irradiating in the adjuvant setting in elderly (> 70 years) HNSCC patients? |
| - - - 1. Do you routinely use alternative does/fractionation schemes in elderly (> 70 years) HNSCC patients? |
| - - - 1. Do you use normal tissue constraints differing form the QUANTEC recommendations in elderly (> 70 years) HNSCC patients? |
| - - - 1. Do you routinely administer chemotherapy in elderly (> 70 years) HNSCC patients, when the established indications are met? |
| - - - 1. Which factors do you routinely incorporate, when deciding about chemotherapy in elderly (> 70 years) HNSCC patients? |
| - - - 1. Which chemotherapy regimen would you recommend for elderly (> 70 years) HNSCC patients in the definitive setting? |
| - - - 1. Which chemotherapy regimen would you recommend for elderly (> 70 years) HNSCC patients in the adjuvant setting, if medically indicated? |
| - - - 1. What is estimated rate of elderly (> 70 years) HNSCC patients that are not treated analog current guidelines (i.e. S3, NCCN, ESTRO-Consensus Guidelines) in your institution for whatever reasons? |
| - - - 1. What is the routine treatment modality for elderly (> 70 years) HNSCC patients in your institution? |
| - - - 1. What is the interval for regular visits during outpatient radiotherapy for elderly (> 70 years) HNSCC patients? |
| - - - 1. What supportive interventions do you routinely apply for elderly (> 70 years) HNSCC patients in your institution? |
| - - - 1. How does the aftercare interval of elderly (> 70 years) HNSCC patients differ from that of younger HNSCC patients? |
| Case primary radiotherapy |
| - - - 1. What treatment strategy would you recommend in this case? |
| - - - 1. What dose would you prescribe for the primary tumor site in this case? |
| - - - 1. Which fractionation scheme would you recommend in this case? |
| Case adjuvant therapy |
| - - - 1. What treatment strategy would you recommend in this case? |
| - - - 1. What dose would you prescribe for the former tumor bed in this case? |
| - - - 1. Which fractionation scheme would you recommend in this case? |
